# Role of Anterior Segment-Optical Coherence Tomography Angiography in Acute Ocular Burns

**DOI:** 10.3390/diagnostics12030607

**Published:** 2022-02-28

**Authors:** Anahita Kate, Sayan Basu

**Affiliations:** 1 The Cornea Institute, Kode Venkatadri Chowdary Campus, LV Prasad Eye Institute, Vijayawada 521134, India; dranahitakate@lvpei.org; 2 The Cornea Institute, Kallam Anji Reddy Campus, LV Prasad Eye Institute, Hyderabad 500034, India; 3 Prof. Brien Holden Eye Research Centre (BHERC), LV Prasad Eye Institute, Hyderabad 500034, India

**Keywords:** acute ocular burns, ocular chemical burns, eye burns, limbal ischemia, optical coherence tomography, angiography, limbal stem cell deficiency

## Abstract

Acute ocular burns have varied manifestations which require prompt diagnosis and management to prevent chronic sequelae. Of these, the detection of limbal ischemia poses a challenge because of the subjective nature of its clinical signs. Anterior segment optical coherence tomography angiography (AS-OCTA) offers an objective method of assessing ischemia in these eyes. This review provides an overview of the technology of AS-OCTA and its applications in acute burns. AS-OCTA generates images by isolating the movement of erythrocytes within blood vessels from sequentially obtained b-scans. Limbal ischemia manifests in these scans as absent vasculature and the extent of ischemia can be quantified using different vessel-related parameters. Of these, the density of vessels is most commonly used and correlates with the severity of the injury. Incorporation of the degree of ischemia in the classification of acute burns has been attempted in animal studies and its extension to human trials may provide an added dimension in determining the final prognosis of these eyes. Thus, AS-OCTA is a promising device that can objectively evaluate limbal ischemia. This will facilitate the identification of patients who will benefit from revascularization therapies and stem cell transplants in acute and chronic ocular burns, respectively.

## 1. Introduction

Ocular burns can present with a myriad of clinical manifestations in the acute phase. The grade of injury and the extent of ocular structures involved often determines the type and severity of the chronic sequelae that ensue. Limbal stem cell deficiency (LSCD) is one such chronic feature that can cause significant visual morbidity due to the corneal scarring and vascularization that occurs in its end stages [1]. The development of this pathology is usually heralded by the presence of limbal ischemia in the acute phase of the disease [2]. The detection and grading of this finding can help identify those individuals who are at a higher risk of developing LSCD. This cohort of patients can then be subjected to regular monitoring which will facilitate early identification and treatment of LSCD to prevent its complications and restore visual function.

However, the diagnosis of ischemia in the acute phase is based on subjective clinical findings which are often difficult to discern in an inflamed eye [2]. Thus, the adoption of diagnostic tools can provide an objective method of confirming ischemia of the limbal vasculature and assessing its progression. An anterior segment optical coherence tomography (AS-OCT) is usually available in most anterior segment practices and provides rapid, high-definition images of sections of the cornea with relative ease [3]. The utility of its angiography feature (AS-OCTA) has recently been explored to detect ischemia in acute burns as it can provide a real time in-vivo image of the limbal and perilimbal vasculature [4,5]. The use of image processing software has also aided in objectively quantifying the degree of vascular changes observed [6]. This review aims to provide an overview of the principles of AS-OCTA and its role in the assessment of limbal ischemia in eyes with acute ocular burns.

## 2. Materials and Methods

A literature search was carried out using the following keywords: “acute ocular burns”, “limbal ischemia”, “optical coherence tomography”, “angiography”, and “limbal stem cell deficiency”; 204 articles were retrieved. Articles were excluded that were not in the English language and were not relevant to our topic. The 53 articles that remained were included in this review.

## 3. Principle and Technology of Optical Coherence Tomography Angiography (OCTA)

### 3.1. Principle

Optical coherence tomography (OCT) measures the signal of backscattered low coherence light incident on the area of interest and uses it to construct sectional and en-face images of different structures of the eye [7,8]. The angiography images are created by obtaining multiple b-scans of the same section over a period of time and assessing the difference in the signal of each scan [7]. This difference is attributed to the movement of erythrocytes within the blood vessels, and by removing the signal from the static surrounding structures, an angiography image is produced by isolating the temporal changes in these dynamic signals [9,10]. Several algorithms are employed to facilitate the same which include the full spectrum or split spectrum amplitude decorrelation angiography (FS or SSADA), optical microangiography (OMAG), intensity ratio analysis (OCTA-RA), etc. They assess the change in the phase of the signal, its amplitude, or both [10].

In SSADA, smaller spectral bands of light are created from the full spectrum and individual analysis is carried out in each band and then averaged [11]. This contrasts with FSADA where the full spectrum is analyzed as a whole. Thus, the SSADA produces images that have a better ratio of signal to noise. However, this is at the expense of axial resolution and this disadvantage is overcome by the OMAG and OCTA-RA algorithms. The former uses both phase and amplitude differences for its images while the latter is independent of the decorrelation principle and assesses the maximum and minimum signal intensity measured between two frames [12,13,14].

### 3.2. Technology

Broadly, the OCT machines can be classified as time-domain and Fourier-domain machines [4]. The current OCTA machines are all Fourier-domain based and consist of the spectral domain {AngioVue (Optovue, Inc., Fremont, CA, USA), Angioscan (Nidek Co., Ltd., Gamagori, Aichi, Japan)} and swept-source OCTA systems {Triton DRI-OCT (Topcon Corporation, Tokyo, Japan), PLEX Elite Prototype 9000 (Carl Zeiss Meditec, Dublin, CA, USA)}. The swept-source OCTAs have a longer wavelength (1050 nm) which improves the depth of structures imaged [15]. This, in combination with a faster speed of scan acquisition (200,000 scans/s), helps improve the resolution and provides a wider field of view [4]. Additionally, the eye tracking software of PLEX Elite is unique to anterior segment devices and can help decrease the artifacts produced [4]. However, the images procured by the AngioVue system have the highest axial and lateral resolution of 5 and 15 μm, respectively [9]. Its speed of acquisition is also the highest at 3–4 s per scan because of which the motion artifacts generated with this device are the least. In comparison with dye-based angiography, both AngioVue and Angioscan machines were found to be comparable, yielding quantifying parameters that correlated well with each other [16,17,18,19]. Although the inter-device values correlate well with each other, the absolute values were found to be greater with AngioVue, implying that these inter-system values cannot be compared with each other [20]. AS-OCTA can not only confirm the presence or absence of vasculature but can also reliably and accurately detect changes in vessel caliber [6].

## 4. Comparison with Fluorescein Angiography (FA) and Indocyanine Green Angiography (ICG)

The most important factor that sets OCTA apart from its counterpart vessel-imaging modalities is its non-invasive nature as it is not dependent on the use of a dye [21,22]. This obviates the injection-associated trauma and dye-associated risks such as nausea, vomiting, and, rarely, anaphylaxis [23,24]. By avoiding the use of an external dye, the time taken for the angiography is significantly decreased with the use of OCTA which is especially desirable in patients with painful disorders such as acute burns. The test can be repeated multiple times without any need for recurrent dye injections. Additionally, the ischemic zones may be obscured by leakage of the dye from the inflamed vessels in these eyes and this disadvantage is circumvented with OCTA [25].

**Figure 1:**
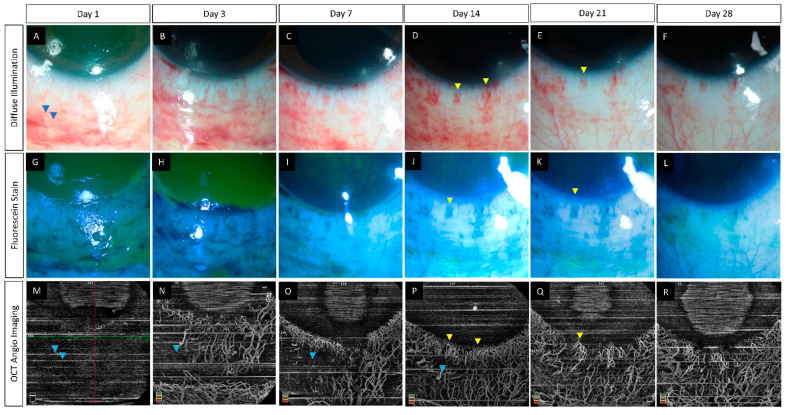
(**A**–**R**) Serial monitoring with slit lamp photographs (without and with fluorescein staining) along with anterior segment optical coherence tomography angiography (AS-OCTA) imaging in an eye with acute chemical burns. (**A**–**L**) These images show the progressive healing of the corneal and conjunctival epithelial defects in the inferior part of the eye which is complete by day 7. (**M**–**R**) AS-OCTA images show a blocked signal in the initial three visits due to the subconjunctival hemorrhages inferiorly (blue arrow heads) with clear delineation of the vasculature following the resolution of the hemorrhages. The hemorrhages abutting the limbus, however, have not affected the vascular signal (yellow arrow heads).

The use of light in the infrared wavelength by the OCTA provides an added advantage as the device can image vessels beneath hemorrhages from superficial conjunctival vessels [6,26]. This is of particular significance in eyes with acute burns as these traumatic sub-conjunctival hemorrhages (SCH) are common and may mask areas of ischemia when viewed via conventional angiographic techniques. However, shadow artifacts can also be seen in AS-OCTA images due to these hemorrhages. This contrast has been depicted in Figure 1 where in the inferior area the vasculature is obscured by the SCH. At the same time in the region abutting the inferior limbus, the vasculature is clear despite the presence of the SCH. One possible explanation for this discrepancy can be the relative position of the SCH when compared to the vessels. Very superficial and large hemorrhages may block the view of all the underlying vessels as opposed to deeper SCH which may allow the imaging of vessels overlying the hemorrhage.

The infrared wavelength of light also allows for better patient comfort and has a good safety profile as well [27]. Localization of the exact depth of a vascular lesion is now feasible because OCTA allows segmentation of different layers of the imaged vessels [16,28,29]. This is in contrast to FA and ICG which generate a composite two-dimensional image. The major drawback of OCTA when compared to the traditional angiography tools lies in its inability to identify the flow characteristics and thus, patterns of leakage, the difference between afferent and efferent systems, etc. cannot be captured with OCTA [30,31]. In the context of ocular burns, this is relevant as images from OCTA may not distinguish vasospasm from true ischemia and thus caution must be exercised during interpretation of OCTA scans. Furthermore, the field of view is larger in FA and ICG and multiple scans would be required by OCTA to cover the same surface area [3,24]. As OCTA images are derived from the motion of blood cells, this process can be confounded by motion originating from other sources, such as movements of the patient’s eyes or head. Although the eye tracking feature of retinal OCTA can counteract these artifacts, its incorporation in anterior segment images has not been completely established.

## 5. Normal Limbal Vasculature

### 5.1. Clinical Anatomy

The limbus is supplied by the anterior roots of the episcleral arterial circle [32]. This circle lies posterior to the limbus and is formed by the branches of the anterior ciliary arteries which in turn are derived from the muscular branches of the ophthalmic artery [32,33]. The arterioles extending from this plexus form a bed of capillaries which then drain into a venous plexus. This venous circle consists of up to three parallel vessels which then become tributaries of episcleral collecting veins [32]. These veins coalesce with the emissary’s veins and exit the surface of the globe [32,33]. Fluorescein angiography studies have revealed a very slow flow in the capillary bed and at the end of the arcade loops [33]. This factor has to be considered during the interpretation of OCTA images as these low flow areas may mimic ischemia.

### 5.2. OCTA of Normal Limbus

#### 5.2.1. Image Acquisition

The OCT machines are devised to image the structure and functional blood flow of the retina and the choroid. To do so, a special adaptor lens attachment is usually required to perform anterior segment angiography; however, the image acquisition can also be carried out by manually adjusting the focus [31,34]. In such cases, the external lens is very close to the patient’s eye and care has to be taken to avoid accidental trauma [3]. Adequate positioning and cooperation of the patient are imperative for acquiring good quality images and keeping motion artifacts to a minimum. In eyes with acute burns, the use of topical anesthetic agents may improve patient comfort, especially in eyes with corneal epithelial defects. Typically, the scan is acquired in all four quadrants separately by asking the patient to look in different directions such that the viewed area is parallel to the device [6]. In Angioscan, a 12 × 9 mm scan is available which provides a panoramic view of the cornea [4]. With the help of this option, the complete limbal vasculature can be screened in one sitting, and then the ischemic areas can be imaged with smaller scan areas (3 × 3 or 6 × 6 mm) to enable their study in greater detail.

**Figure 2:**
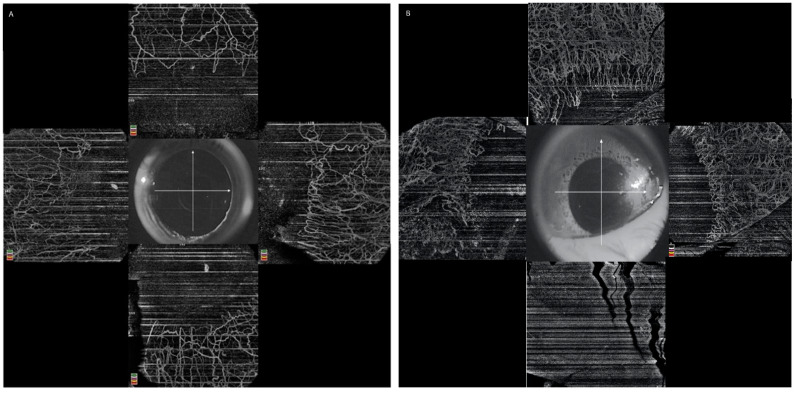
Collage of images depicting the role of the infrared image in anterior segment optical coherence tomography angiography (AS-OCTA) (**A**) AS-OCTA image of a normal eye with good exposure of the structures on the infrared image (**B**) Image of an eye with chemical injury showing the effect of an updrawn lower lid mimicking ischemia of the inferior limbus and sclera.

It is essential to assess a few aspects before proceeding with the interpretation of the image. The infrared image of the cornea provided with the angiography scan gives a good idea of the position of the adnexal structures at the time of scan procurement. Checking this image is vital, as an updrawn lower lid due to traction on the upper lid can cover the lower limbus and sclera. This may appear as a non-perfused area on the OCTA scan (Figure 2). The signal strength provided by most OCTA is calibrated for retinal evaluation, though a few programs are optimized to assess the same for anterior segment scans [26]. An alternative method of assessing the quality of the image has been proposed by Ang et al. based on the delineation of vessels [35]. It consists of five grades ranging from no discernible vessels to excellent delineation of the vessels. The presence of motion or projection artifacts must also be checked as they will affect the quantitative analysis and, if required, the scans may have to be repeated [10,36]. Projection artifacts arise in deeper layers when the flow from superficial vessels gets superimposed on them and this may lead to underestimation of the degree of ischemia.

#### 5.2.2. Normal Limbal Vasculature on OCTA

**Figure 3:**
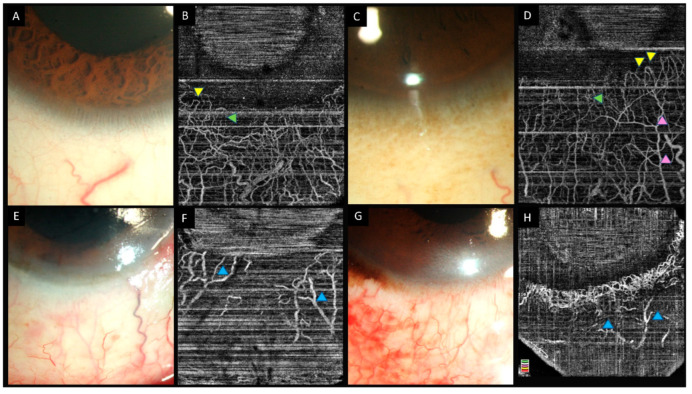
(**A**–**D**) Collage of images of the slit lamp and anterior segment optical coherence tomography angiography images of eyes with normal limbal and perilimbal vasculature. Hairpin loop vessels are noted at the corneal end of the limbus (yellow arrowheads). A combination of both Y-shaped (pink arrowheads) and radial vessels (green arrowheads) are noted in the angiography images. (**E**–**H**) Slit lamp and OCTA images in eyes with chemical injury illustrating ischemic zones in both angiographies with distortion of the limbal vasculature (blue arrowheads).

The normal limbal vessels appear as hairpin loops that extend 2–3 mm into the corneal surface and form a peripheral marginal arcade (Figure 3). Segmentation studies have revealed superficial vessels which extend radially with smaller vessels uniformly among them [28]. On the other hand, deeper vessels showed a sectoral pattern with dense intrascleral plexus near the limbus that becomes sparse while moving towards the periphery [28]. A Y-shaped flow pattern has also been seen in these deep layers [28].

#### 5.2.3. Qualitative Assessment of Limbal Vasculature

**Figure 4:**
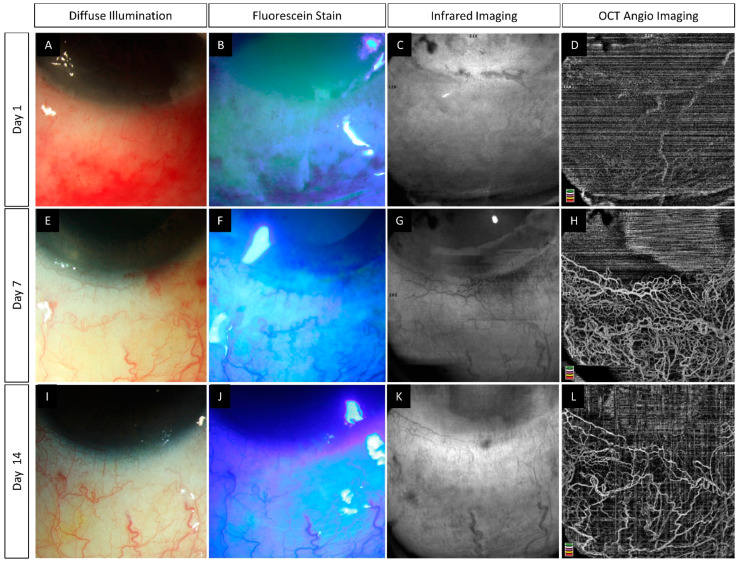
(**A**–**L**) Slit lamp photographs taken without and with fluorescein staining along with infrared and anterior segment optical coherence tomography angiography (AS-OCTA) images depicting the course of healing in an eye with acute chemical burns. An inferior corneal and conjunctival epithelial defect is noted (**A**,**B**) at presentation which heals over the course of two weeks (**E**,**F**,**I**,**J**). (**D**) AS-OCTA image taken at presentation shows a lack of vessels in the inferior half of the eye. (**H**,**L**) A progressive increase is noted in the number of visible vessels suggesting the presence of vasospasm in the initial images which has reversed in the subsequent visits.

**Figure 5:**
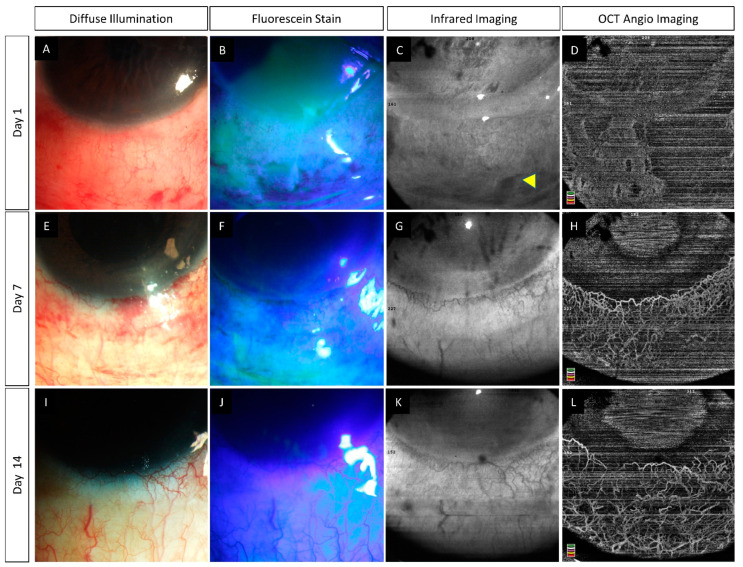
(**A**–**L**) Slit lamp photographs captured without and with fluorescein staining along with infrared and anterior segment optical coherence tomography angiography (AS-OCTA) images in an eye with acute chemical burns. An inferior corneal and conjunctival epithelial defect is present at the first visit (**A**,**B**). Healing of the corneal defect is observed within one week (**E**,**F**) while the conjunctival defect takes two weeks for the same (**I**,**J**). (**C**,**G**,**K**) Sub-conjunctival hemorrhages are clearly delineated on the infrared image taken on day 1 (yellow arrowhead) and its resolution is noted in the subsequent visits. (**D**,**H**,**L**) AS-OCTA image taken at presentation depicts partial lack of vascular signal which gradually recovers over the subsequent follow-up visits.

The severity of ischemia with acute burns can be assessed quantitatively or qualitatively. The presence or absence of ischemia is determined by the areas that appear dark on angiography. As the quantitative assessment requires post-acquisition processing with specialized software, implementation of the same in routine clinical practice is cumbersome. This drawback can be circumvented with the inclusion of customized programs for anterior segment analysis within the OCTA machines. Caution must be exercised before terming the mere absence of notable vasculature as ischemia as these dark areas may manifest due to vasospasm as well. Serial monitoring of such cases will help ascertain the underlying cause, as vasospasm is reversible and visible vessels can be observed on the follow-up scans (Figure 4 and Figure 5). The intensity of signal measured has also been used to assess the velocity of blood, with brighter signals indicating the rapid transit of the red blood cells [37].

#### 5.2.4. Quantitative Assessment of Limbal Vasculature

The scans obtained from the OCTA systems are exported for analysis into image processing software such as Fiji (National Institutes of Health, Bethesda, MD, USA) and MATLAB (MathWorks, Inc., Natick, MA, USA) software [6,20,38]. Patel et al. helped establish a standardized methodology for the quantification of images procured by an AS-OCTA [6]. An area of interest is highlighted from the OCTA image and converted to a greyscale image. A manual inspection is usually required to detect and remove artifacts. Automated reduction of noise is also feasible through the software mentioned above by the thresholding of a signal greater than a pre-defined value [6,20,39]. This image is subsequently binarized by the software wherein the vessel is assigned one color and the background another. A skeletonized version of the image is then generated by reducing the width of the vessel down to one pixel [40]. Although there is no customized segmentation software for anterior segment imaging, the use of retinal segmentation tools has been described for splitting the limbal vasculature into superficial and deep layers [28,41]. Vessels up to a depth of 200 μm are considered superficial while those beyond 200 μm are deemed as deep vasculature.

Various indices have been described for corneal vasculature of which vessel density (VD), vessel length density (VLD), vessel diameter index (VDI), and fractal dimension are most commonly used for limbal vasculature quantification. Table 1 summarizes the different parameters, the way they are computed, and the ranges reported from different studies. VD has been utilized more frequently for studies on limbal vasculature and represents the overall extent of vascularization while VLD and VDI estimate the caliber of the vessels. Fractal dimension is an interesting factor that quantifies the complexity of branching and can have values ranging from 0 to 2 with values that are closer to 2 having greater tortuosity and branching [42,43,44]. The direct estimation of the extent of limbal disruption due to chemical injury as seen on OCTA has also been divided into four grades (0: no limbal disruption, 1: <25%, 2: 25–50%, and 3: >50%) [35].

OCTA in retinal imaging utilizes parameters that are specific to the assessment of ischemia such as flow void and flow index which evaluate the area of ischemia and velocity of blood flow, respectively [45,46]. The vessel perimeter index can also detect early ischemia and the inclusion of these indices can better quantify the degree of ischemia and understand its changes with varying severity of the injury. [47,48] The pattern of reperfusion that occurs following interventions such as tenonplasty can also be studied with these factors.

## 6. OCTA in Acute Ocular Burns

### 6.1. Role of AS-OCTA

As previously discussed, relying only on clinical features for judging the extent and progression of limbal ischemia may not provide an accurate picture as surface inflammation and edema may alter the clinical presentation. Kam et al. studied this clinical discrepancy and found it existed across all levels of expertise [2]. The assessment of the extent of limbal ischemia is of utmost importance as it not only predicts the risk of development of LSCD in the future but also dictates the need for surgical interventions in the acute phase, such as tenonplasty. This procedure involves the advancement of Tenon’s capsule to help re-perfuse the ischemic areas. The use of FA has been described to determine the area of limbal or scleral ischemia that will require the procedure, and the same purpose can be extrapolated to OCTA [49,50]. Furthermore, the extent of ischemia can be used to prognosticate the outcome in terms of vision and development of LSCD.

**Figure 6:**
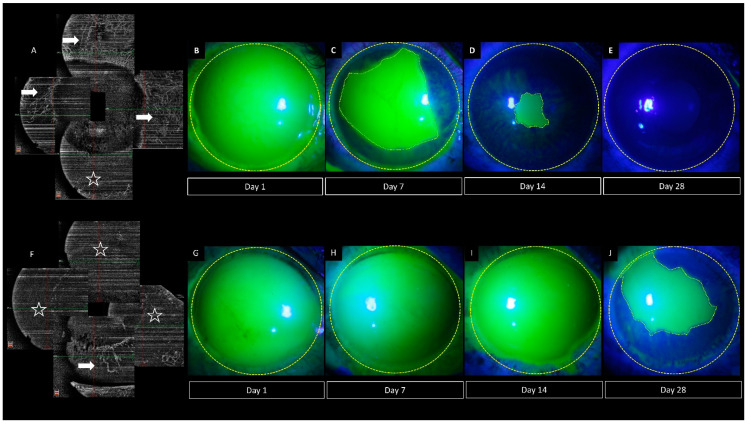
Collage of images depicting different rates of healing of total corneal epithelial defects in two cases of acute ocular burns. (**A**,**F**) Optical coherence tomography angiography images of both cases, showing greater ischemia in the eye in the lower panel when compared to the upper panel (white arrows denote the perfused areas while the white stars denote the non-perfused zones). The corresponding epithelial defect of the patient in the upper panel healed faster with a completely epithelialized surface at the 1-month visit (**B**–**E**) when compared to the healing of the patient in the lower panel with a persistent defect at the end of 1 month of follow up (**G**–**J**).

The chemical agent itself and the associated inflammation lead to disruption of normal epithelial healing in acute ocular burns [51,52]. Limbal and scleral ischemia can contribute to this delayed healing as seen in Figure 6 where both patients have total epithelial defects at presentation. However, the patient in the upper panel has lesser ischemia on OCTA with faster healing of the epithelial defect. The use of an AS-OCTA can help identify these cases where slow epithelialization is anticipated and help determine the need for early intervention with either an amniotic membrane or tarsorrhaphy.

**Figure 7:**
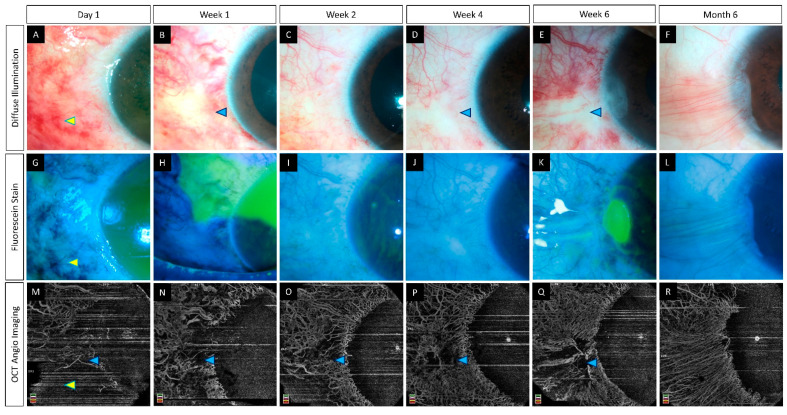
(**A**–**R**) Serial imaging with slit lamp photographs (without and with fluorescein staining) and optical coherence tomography angiography imaging in an eye with acute chemical burns. (**A**–**L**) These images depict a total corneal epithelial defect with an adjacent conjunctival defect at presentation, which decreased in size and ultimately healed after 2 weeks. (**M**–**R**) The corresponding angiography images illustrate an initial masked signal due to the sub-conjunctival hemorrhages (yellow arrowheads) which recovered with the resolution of the hemorrhages. Persistent ischemia is noted in the limbal area abutting the cornea (blue arrowheads) with epithelial healing issues in the adjacent cornea at the 6th week visit. A partial LSCD is noted in the same area at the 6th month follow-up (**F**).

Epithelial instability with repeated breakdowns usually occurs in the presence of an unhealthy limbus [53,54]. However, these features can also manifest without any obvious signs of LSCD. An AS-OCTA can help detect the underlying limbal stem cell disruption in these eyes. This has been illustrated in Figure 7 where a focal area of epithelial defect is noted adjacent to the non-perfused area on AS-OCTA.

### 6.2. Pre-Clinical Studies

In a rabbit model where the chemical injury was induced in a graded manner in different quadrants of the eye, the changes in limbal vasculature were studied over a period of 1 month using AS-OCTA [55]. The fellow eyes were uninvolved and taken as controls. The authors found a significant decrease in the vessel density in the affected eyes when compared to the controls. This change was evident from day 1 of the injury, implying that the OCTA systems are sensitive enough to detect changes in the acute phase. Additionally, the degree of decrease in vessel density correlated with the severity of the induced burn leading the authors to propose a classification system that included these OCTA values. This correlation also suggests that the OCTA values may also be used in isolation as objective measures to predict prognosis. Further clinical studies in humans implementing such classifications can help determine the prognosis and outcomes associated with varying grades of limbal ischemia on OCTA. At 1 month, the eyes with the chemical injury had a significantly higher vessel density than the fellow eyes. This increase was attributed to the formation of a pannus over the corneal surface. Luisi et al. described with OCTA and FA, the limbal and corneal vascular changes in mice with induced chemical injury [56]. They found both angiography modalities yielded similar results and detected nascent vessels in the limbal area as early as 4 days following the injury.

### 6.3. Clinical Studies

In a study by Fung et al., the extent of ischemia as seen on clinical examination was found to be underestimated when compared to that measured by OCTA [26]. Similarly, in a study comparing two OCTA systems for assessment of limbal ischemia, the authors also found a good inter-grader agreement for both OCTAs, while the same was not present for findings on slit lamp examination [35]. Furthermore, the ischemic areas on OCTA were found to be significantly lesser than the measured area of conjunctival defects. The above findings illustrate the discordance between clinical signs and true ischemia. The authors also demonstrated the use of the segmentation feature to understand the degree of ischemia by depicting differential ischemia in the superficial conjunctival and the deeper intrascleral vessels [26]. Although there are reports of improvement in perfusion of the limbal area with time, which is not clinically evident but can be detected on FA, the same findings were not seen on longitudinal assessment with OCTA [26,57].

A strong positive correlation has been described between the extent of limbal ischemia and visual acuity at 3 months [26]. In addition, the development of LSCD occurred in the same areas of ischemia seen in the acute phase [26]. Ang et al. failed to demonstrate a similar correlation between limbal staining and the development of LSCD [35]. This may aid clinicians to predict the degree of LSCD that will occur and thus determine the need for autologous or allogenic stem cell transplants well in advance. A summary of the various studies on the role of AS-OCTA in acute chemical injury has been presented in Table 2.

## 7. Future Directions

The angiography feature of OCT has immense potential in confirming the presence of and assessing the progression of limbal ischemia in eyes with acute ocular burns. However, the technology is still nascent with several facets that can be improved upon. This includes the incorporation of eye tracking software which will help decrease motion artifacts and facilitate reliable comparison between scans taken over a period of time. As most of the current designs of AS-OCTA are extensions of their retinal counterparts, the software algorithms are not customized for anterior segment imaging. The development of inbuilt tools that will quantify corneal and limbal vasculature changes will provide a standardized platform for assessing eyes with acute and chronic afflictions of ocular burns. Furthermore, there is also a need for OCTA devices to be able to distinguish between areas of low flow and true ischemia.

With the introduction of ultra-widefield OCTA, it is now possible to have a panoramic view of the entire 360 degrees of the limbal vessels [58]. This will provide a bird’s eye view of the extent of ischemia and may serve as a screening tool in eyes with acute burns. Additionally, the combination of both full and split spectrum algorithms within a single device is being explored which significantly improves the resolution of the image acquired and provides an option for eye tracking [59]. The machine also helps gauge the direction of flow within corneal and limbal vessels and this feature may provide a unique perspective of the ocular surface vasculature in various pathologies. Segmentation software that is specific to the cornea and limbus with pre-set thickness cut-offs will help standardize the image outputs and the quantitative measurements.

Studies assessing limbal ischemia in different grades of acute ocular burns can help understand the correlation of this finding with the progression of epithelial healing and the development of chronic sequelae such as LSCD. The establishment of normative data for quantitative indices of limbal vasculature will provide baseline values and help assess the degree of change with an acute burn injury. A majority of the literature on use of AS-OCTA in acute burns has focused on the variation in vessel density for the assessment of limbal vessels, and change in parameters such as vessel branch length, width, etc. is yet to be studied. The incorporation of gradations of these values within the existing classifications will aid in formulating a better prognosticating system for acute ocular burns.

## 8. Summary

Evaluation of limbal ischemia is critical in eyes with acute burns to administer adequate care and to determine the long-term prognosis. Clinical examination alone is insufficient to assess the presence and extent of ischemia. AS-OCTA provides an objective and reliable method of not only confirming ischemia of the limbal vessels but also quantifying the same. The images are captured via a rapid and non-contact process and do not rely on the use of a dye. Various parameters have been described for the quantification of the ischemia of which vessel density has been used most frequently. A change in density can be detected within 24 h of presentation and the degree of change correlates with the severity of the injury. Serial monitoring is required to differentiate ischemia from vasospasm. Further studies are necessary to establish diagnostic values for indices assessing limbal ischemia which will facilitate the incorporation of this device into clinical practice for the management of ocular burns.

## Figures and Tables

**Table 1:** Summary of various vessel-related parameters used for quantifying limbal vasculature and their reported ranges.

Indices	Method of Calculation [14,15]	Range [28,41]	
		***Superficial layer***	***Deep layer***
Vessel density (%)	Area occupied by the vessels/ Total area of the background	24–31	21–29
Vessel length density (%)	Skeletonized vessel pixel area/ total pixel count	4–5	3.5–4.5
Vessel diameter index	Total vessel area in binarized image/ Total vessel area in the skeletonized image	10–17	10–18
Fractal dimension	Box counting method	1.3–1.5	1.2–1.5

**Table 2:** A review of studies on the use of AS-OCTA in acute ocular burns.

Author (Year)	Study Population (Eyes)	Device Used	Duration of Follow up (Months)	Outcome
Fung et al., (2019) [26]	Humans (15)	AngioVue	1	(i) Clinically determined area of ischemia is underestimated when compared to estimated ischemia on OCTA. (ii) Area of ischemia on OCTA correlates with visual acuity at 3 months.
Ang et al., (2021) [35]	Humans (10)	(i) AngioVue (ii) Plex Elite	3	(i) Good agreement between the two devices for the measurement of VD. (ii) OCTA is more reliable than clinical examination for the assessment of limbal ischemia. (iii) Area of ischemia may predict the future development of LSCD.
Tey et al., (2021) [55]	Rabbits (12)	Spectralis-domain OCT (Nidek Co., Ltd., Gamagori, Aichi, Japan)	1	(i) Decrease in VD correlated with the severity of the induced injury. (ii) Proposed classification system which incorporates limbal ischemia as estimated by OCTA.
Luisi et al., (2021) [56]	Mice (12)	Envisu R2200 (Bioptigen, Durham, NC)	0.5	(i) Both OCTA and FA angiography modalities yielded similar results. (ii) Detection of neovascularization in the limbal area as early as 4 days following the injury.

OCTA—optical coherence tomography angiography, VD—vessel density, FA—fluorescein angiography.

## Data Availability

This is a review article, and no new data were analyzed in this study. Data sharing is not applicable to this article.

## References

[1] Haagdorens M., Van Acker S.I., Van Gerwen V., Ní Dhubhghaill S., Koppen C., Tassignon M.-J., Zakaria N. (2016). Limbal Stem Cell Deficiency: Current Treatment Options and Emerging Therapies. Stem Cells Int..

[2] Kam K.W., Patel C.N., Nikpoor N., Yu M., Basu S. (2019). Limbal Ischemia: Reliability of Clinical Assessment and Implications in the Management of Ocular Burns. Indian J. Ophthalmol..

[3] Ayres M., Smallwood R., Brooks A.M., Chan E., Fagan X. (2019). Anterior Segment Optical Coherence Tomography Angiography. J. Vis. Commun. Med..

[4] Lee W.D., Devarajan K., Chua J., Schmetterer L., Mehta J.S., Ang M. (2019). Optical Coherence Tomography Angiography for the Anterior Segment. Eye Vis..

[5] Cozzi M., Staurenghi G., Invernizzi A. (2020). Anterior Segment and Ocular Adnexa OCT Angiography. Ophthalmology.

[6] Patel C.N., Antony A.K., Kommula H., Shah S., Singh V., Basu S. (2020). Optical Coherence Tomography Angiography of Perilimbal Vasculature: Validation of a Standardised Imaging Algorithm. Br. J. Ophthalmol..

[7] Fujimoto J.G., Pitris C., Boppart S.A., Brezinski M.E. (2000). Optical Coherence Tomography: An Emerging Technology for Biomedical Imaging and Optical Biopsy. Neoplasia.

[8] Borrelli E., Parravano M., Sacconi R., Costanzo E., Querques L., Vella G., Bandello F., Querques G. (2020). Guidelines on Optical Coherence Tomography Angiography Imaging: 2020 Focused Update. Ophthalmol. Ther..

[9] Munk M.R., Giannakaki-Zimmermann H., Berger L., Huf W., Ebneter A., Wolf S., Zinkernagel M.S. (2017). OCT-Angiography: A Qualitative and Quantitative Comparison of 4 OCT-A Devices. PLoS ONE.

[10] Onishi A.C., Fawzi A.A. (2019). An Overview of Optical Coherence Tomography Angiography and the Posterior Pole. Ther. Adv. Ophthalmol..

[11] Jia Y., Tan O., Tokayer J., Potsaid B., Wang Y., Liu J.J., Kraus M.F., Subhash H., Fujimoto J.G., Hornegger J. (2012). Split-Spectrum Amplitude-Decorrelation Angiography with Optical Coherence Tomography. Opt. Express.

[12] An L., Wang R.K. (2008). In Vivo Volumetric Imaging of Vascular Perfusion within Human Retina and Choroids with Optical Micro-Angiography. Opt. Express.

[13] Wang R.K., An L., Francis P., Wilson D.J. (2010). Depth-Resolved Imaging of Capillary Networks in Retina and Choroid Using Ultrahigh Sensitive Optical Microangiography. Opt. Lett.

[14] Stanga P.E., Tsamis E., Papayannis A., Stringa F., Cole T., Jalil A. (2016). Swept-Source Optical Coherence Tomography Angio^TM^ (Topcon Corp, Japan): Technology Review. Dev. Ophthalmol..

[15] Ang M., Cai Y., Tan A.C.S. (2016). Swept Source Optical Coherence Tomography Angiography for Contact Lens-Related Corneal Vascularization. J. Ophthalmol..

[16] Devarajan K., Di Lee W., Ong H.S., Lwin N.C., Chua J., Schmetterer L., Mehta J.S., Ang M. (2019). Vessel Density and En-Face Segmentation of Optical Coherence Tomography Angiography to Analyse Corneal Vascularisation in an Animal Model. Eye Vis..

[17] Ang M., Cai Y., MacPhee B., Sim D.A., Keane P.A., Sng C.C.A., Egan C.A., Tufail A., Larkin D.F., Wilkins M.R. (2016). Optical Coherence Tomography Angiography and Indocyanine Green Angiography for Corneal Vascularisation. Br. J. Ophthalmol..

[18] Hong J., Zhu W., Zhuang H., Xu J., Sun X., Le Q., Li G., Wang Y. (2010). In Vivo Confocal Microscopy of Conjunctival Goblet Cells in Patients with Sjogren’s Syndrome Dry Eye. Br. J. Ophthalmol..

[19] Stanzel T.P., Devarajan K., Lwin N.C., Yam G.H., Schmetterer L., Mehta J.S., Ang M. (2018). Comparison of Optical Coherence Tomography Angiography to Indocyanine Green Angiography and Slit Lamp Photography for Corneal Vascularization in an Animal Model. Sci. Rep..

[20] Ang M., Devarajan K., Das S., Stanzel T., Tan A., Girard M., Schmetterer L., Mehta J.S. (2018). Comparison of Anterior Segment Optical Coherence Tomography Angiography Systems for Corneal Vascularisation. Br. J. Ophthalmol..

[21] Kuckelkorn R., Remky A., Wolf S., Reim M., Redbrake C. (1997). Video Fluorescein Angiography of the Anterior Eye Segment in Severe Eye Burns. Acta Ophthalmol. Scand..

[22] Cai Y., Alio Del Barrio J.L., Wilkins M.R., Ang M. (2017). Serial Optical Coherence Tomography Angiography for Corneal Vascularization. Graefes Arch. Clin. Exp. Ophthalmol..

[23] Xu K., Tzankova V., Li C., Sharma S. (2016). Intravenous Fluorescein Angiography-Associated Adverse Reactions. Can. J. Ophthalmol..

[24] Anijeet D.R., Zheng Y., Tey A., Hodson M., Sueke H., Kaye S.B. (2012). Imaging and Evaluation of Corneal Vascularization Using Fluorescein and Indocyanine Green Angiography. Investig. Ophthalmol. Vis. Sci..

[25] Ang M., Tan A.C.S., Cheung C.M.G., Keane P.A., Dolz-Marco R., Sng C.C.A., Schmetterer L. (2018). Optical Coherence Tomography Angiography: A Review of Current and Future Clinical Applications. Graefes Arch. Clin. Exp. Ophthalmol..

[26] Fung S.S.M., Stewart R.M.K., Dhallu S.K., Sim D.A., Keane P.A., Wilkins M.R., Tuft S.J. (2019). Anterior Segment Optical Coherence Tomographic Angiography Assessment of Acute Chemical Injury. Am. J. Ophthalmol..

[27] Shu X., Beckmann L., Zhang H.F. (2017). Visible-Light Optical Coherence Tomography: A Review. J. Biomed. Opt..

[28] Akagi T., Uji A., Huang A.S., Weinreb R.N., Yamada T., Miyata M., Kameda T., Ikeda H.O., Tsujikawa A. (2018). Conjunctival and Intrascleral Vasculatures Assessed Using Anterior Segment Optical Coherence Tomography Angiography in Normal Eyes. Am. J. Ophthalmol..

[29] Giarratano Y., Bianchi E., Gray C., Morris A., MacGillivray T., Dhillon B., Bernabeu M.O. (2020). Automated Segmentation of Optical Coherence Tomography Angiography Images: Benchmark Data and Clinically Relevant Metrics. Transl. Vis. Sci. Technol..

[30] Siddiqui Y., Yin J. (2019). Anterior Segment Applications of Optical Coherence Tomography Angiography. Semin. Ophthalmol..

[31] Luo M., Li Y., Zhuo Y. (2021). Advances and Current Clinical Applications of Anterior Segment Optical Coherence Tomography Angiography. Front. Med..

[32] Meyer P.A. (1989). The Circulation of the Human Limbus. Eye.

[33] Wang Y., Chodosh J. (2019). Angiography of the Limbus and Cornea. Int. Ophthalmol. Clin..

[34] Brunner M., Romano V., Steger B., Vinciguerra R., Lawman S., Williams B., Hicks N., Czanner G., Zheng Y., Willoughby C.E. (2018). Imaging of Corneal Neovascularization: Optical Coherence Tomography Angiography and Fluorescence Angiography. Investig. Ophthalmol. Vis. Sci..

[35] Ang M., Foo V., Ke M., Tan B., Tong L., Schmetterer L., Mehta J.S. (2021). Role of Anterior Segment Optical Coherence Tomography Angiography in Assessing Limbal Vasculature in Acute Chemical Injury of the Eye. Br. J. Ophthalmol..

[36] Li X.-X., Wu W., Zhou H., Deng J.-J., Zhao M.-Y., Qian T.-W., Yan C., Xu X., Yu S.-Q. (2018). A Quantitative Comparison of Five Optical Coherence Tomography Angiography Systems in Clinical Performance. Int. J. Ophthalmol..

[37] Pichi F., Roberts P., Neri P. (2019). The Broad Spectrum of Application of Optical Coherence Tomography Angiography to the Anterior Segment of the Eye in Inflammatory Conditions: A Review of the Literature. J. Ophthalmic. Inflamm. Infect..

[38] Ang M., Baskaran M., Werkmeister R.M., Chua J., Schmidl D., Aranha dos Santos V., Garhöfer G., Mehta J.S., Schmetterer L. (2018). Anterior Segment Optical Coherence Tomography. Prog. Retin. Eye Res..

[39] Varma S., Shanbhag S.S., Donthineni P.R., Mishra D.K., Singh V., Basu S. (2021). High-Resolution Optical Coherence Tomography Angiography Characteristics of Limbal Stem Cell Deficiency. Diagnostics.

[40] Durbin M.K., An L., Shemonski N.D., Soares M., Santos T., Lopes M., Neves C., Cunha-Vaz J. (2017). Quantification of Retinal Microvascular Density in Optical Coherence Tomographic Angiography Images in Diabetic Retinopathy. JAMA Ophthalmol..

[41] Bostanci Ceran B., Ozates S., Arifoglu H.B., Tasindi E. (2021). Changes in Limbal Optical Coherence Tomography Angiography Outcomes in Patients With Overnight Contact Lens Wear. Eye Contact Lens.

[42] Huang P.-W., Lee C.-H. (2009). Automatic Classification for Pathological Prostate Images Based on Fractal Analysis. IEEE Trans. Med. Imaging.

[43] Kim A.Y., Chu Z., Shahidzadeh A., Wang R.K., Puliafito C.A., Kashani A.H. (2016). Quantifying Microvascular Density and Morphology in Diabetic Retinopathy Using Spectral-Domain Optical Coherence Tomography Angiography. Investig. Ophthalmol. Vis. Sci..

[44] Reif R., Qin J., An L., Zhi Z., Dziennis S., Wang R. (2012). Quantifying Optical Microangiography Images Obtained from a Spectral Domain Optical Coherence Tomography System. Int. J. Biomed. Imaging.

[45] Zhang Q., Zheng F., Motulsky E.H., Gregori G., Chu Z., Chen C.-L., Li C., de Sisternes L., Durbin M., Rosenfeld P.J. (2018). A Novel Strategy for Quantifying Choriocapillaris Flow Voids Using Swept-Source OCT Angiography. Investig. Ophthalmol. Vis. Sci..

[46] Hagag A.M., Gao S.S., Jia Y., Huang D. (2017). Optical Coherence Tomography Angiography: Technical Principles and Clinical Applications in Ophthalmology. Taiwan J. Ophthalmol..

[47] Yao X., Alam M.N., Le D., Toslak D. (2020). Quantitative Optical Coherence Tomography Angiography: A Review. Exp. Biol. Med..

[48] Chu Z., Lin J., Gao C., Xin C., Zhang Q., Chen C.-L., Roisman L., Gregori G., Rosenfeld P.J., Wang R.K. (2016). Quantitative Assessment of the Retinal Microvasculature Using Optical Coherence Tomography Angiography. J. Biomed. Opt..

[49] Tabatabaei S.A., Soleimani M., Mirshahi R., Zandian M., Ghasemi H., Hashemian M.N., Ghomi Z. (2017). Selective Localized Tenonplasty for Corneal Burns Based on the Findings of Ocular Surface Fluorescein Angiography. Cornea.

[50] Kuckelkorn R., Redbrake C., Reim M. (1997). Tenonplasty: A New Surgical Approach for the Treatment of Severe Eye Burns. Ophthalmic. Surg. Lasers.

[51] Sharma N., Kaur M., Agarwal T., Sangwan V.S., Vajpayee R.B. (2018). Treatment of Acute Ocular Chemical Burns. Surv. Ophthalmol..

[52] Bizrah M., Yusuf A., Ahmad S. (2019). An Update on Chemical Eye Burns. Eye.

[53] Sangwan V.S. (2001). Limbal Stem Cells in Health and Disease. Biosci. Rep..

[54] Le Q., Xu J., Deng S.X. (2018). The Diagnosis of Limbal Stem Cell Deficiency. Ocul. Surf..

[55] Tey K.Y., Gan J., Foo V., Tan B., Ke M.Y., Schmetterer L., Mehta J.S., Ang M. (2021). Role of Anterior Segment Optical Coherence Tomography Angiography in the Assessment of Acute Chemical Ocular Injury: A Pilot Animal Model Study. Sci. Rep..

[56] Luisi J., Kraft E.R., Giannos S.A., Patel K., Schmitz-Brown M.E., Reffatto V., Merkley K.H., Gupta P.K. (2021). Longitudinal Assessment of Alkali Injury on Mouse Cornea Using Anterior Segment Optical Coherence Tomography. Transl. Vis. Sci. Technol..

[57] Rocha de Lossada C., Pagano L., Gadhvi K.A., Borroni D., Figueiredo G., Kaye S., Romano V. (2020). Persistent Loss of Marginal Corneal Arcades after Chemical Injury. Indian J. Ophthalmol..

[58] Fard A., Wang Y., Kost J., Sha P., Mitra R., Leahy C. (2020). Ultra-Widefield Anterior Segment OCT Angiography for Visualization of Iris, Limbus and Sclera. Investig. Ophthalmol. Vis. Sci..

[59] Mazlin V., Xiao P., Scholler J., Irsch K., Grieve K., Fink M., Boccara A.C. (2020). Real-Time Non-Contact Cellular Imaging and Angiography of Human Cornea and Limbus with Common-Path Full-Field/SD OCT. Nat. Commun..

